# Basophil-Derived IL-4 and IL-13 Protect Intestinal Barrier Integrity and Control Bacterial Translocation during Malaria

**DOI:** 10.4049/immunohorizons.2300084

**Published:** 2024-05-23

**Authors:** Nora Céspedes, Abigail M. Fellows, Erinn L. Donnelly, Hannah L. Kaylor, Taylor A. Coles, Ryan Wild, Megan Dobson, Joseph Schauer, Judy Van de Water, Shirley Luckhart

**Affiliations:** * Department of Entomology, Plant Pathology and Nematology, University of Idaho, Moscow, ID; † Department of Biological Sciences, University of Idaho, Moscow, ID; ‡ Division of Rheumatology, Allergy and Clinical Immunology, University of California, Davis, CA

## Abstract

Our previous work demonstrated that basophils regulate a suite of malaria phenotypes, including intestinal mastocytosis and permeability, the immune response to infection, gametocytemia, and parasite transmission to the malaria mosquito *Anopheles stephensi*. Given that activated basophils are primary sources of the regulatory cytokines IL-4 and IL-13, we sought to examine the contributions of these mediators to basophil-dependent phenotypes in malaria. We generated mice with basophils depleted for IL-4 and IL-13 (baso IL-4/IL-13 (−)) and genotype controls (baso IL-4/IL-13 (+)) by crossing mcpt8-Cre and *Il4/Il13*^fl/fl^ mice and infected them with *Plasmodium yoelii yoelii* 17XNL. Conditional deletion was associated with ileal mastocytosis and mast cell (MC) activation, increased intestinal permeability, and increased bacterial 16S levels in blood, but it had no effect on neutrophil activation, parasitemia, or transmission to *A. stephensi*. Increased intestinal permeability in baso IL-4/IL-13 (−) mice was correlated with elevated plasma eotaxin (CCL11), a potent eosinophil chemoattractant, and increased ileal MCs, proinflammatory IL-17A, and the chemokines MIP-1α (CCL3) and MIP-1β (CCL4). Blood bacterial 16S copies were positively but weakly correlated with plasma proinflammatory cytokines IFN-γ and IL-12p40, suggesting that baso IL-4/IL-13 (−) mice failed to control bacterial translocation into the blood during malaria infection. These observations suggest that basophil-derived IL-4 and IL-13 do not contribute to basophil-dependent regulation of parasite transmission, but these cytokines do orchestrate protection of intestinal barrier integrity after *P. yoelii* infection. Specifically, basophil-dependent IL-4/IL-13 control MC activation and prevent infection-induced intestinal barrier damage and bacteremia, perhaps via regulation of eosinophils, macrophages, and Th17-mediated inflammation.

## Introduction

Malaria is a vector-borne parasitic disease transmitted by *Anopheles* mosquitoes and caused by infection with *Plasmodium* spp. In 2021, the World Health Organization reported 247 million cases and 619,000 malaria deaths, mainly in children under the age of 5 (1). Numerous studies have indicated that malaria predisposes individuals to concomitant bacteremia, resulting in an increased risk of mortality (2–6).

We previously demonstrated in mouse and nonhuman primate malaria models that intestinal permeability and bacteremia are associated with increased intestinal mast cell (MC) influx or mastocytosis (7–9). MC-deficient mice with malaria exhibited reduced gut permeability and bacteremia relative to controls (8), supporting the involvement of MCs in malaria-induced gut barrier disruption. Moreover, intestinal mastocytosis and bacteremia in *Plasmodium yoelii yoelii* 17XNL–infected mice were associated with the early appearance of IL-4, IL-18, MC protease (Mcpt)4, and basophils in plasma, followed by elevated levels of IgE, IL-9, IL-13, and Mcpt1, mediators that can maintain and enhance MC activation (9).

Basophils share phenotypic similarities with MCs and are also involved in inflammation, having been associated with a protective role against tissue damage and pathology following helminth infection (10). In experimental murine colitis, basophils play a crucial role in regulating T cell responses and limiting disease (11), and they have a proinflammatory role in persistent intestinal inflammation, such as ulcerative colitis (12). Upon activation, basophils degranulate, resulting in the release of histamine, chemokines, and cytokines including IL-4 and IL-13 (13). These cytokines are recognized as markers of a type 2 inflammatory response, and they share a receptor, a heterodimer consisting of an IL-4Rα and an IL-13Rα1 subunit (14).

In mouse malaria, we have observed that *P. y. yoelii* 17XNL–infected, basophil-depleted mice exhibited significantly increased intestinal permeability throughout the course of infection (15), whereas mice with basophils lacking IL-18R [baso IL-18R (−)] presented increased intestinal permeability only at day 4 postinfection (PI) (16) relative to their genotype controls. Moreover, both basophil-depleted and baso IL-18R (−) mice exhibited increased ileal MCs at 8 and 10 d PI, respectively (15, 16), suggesting that basophils play a protective role in homeostasis of the intestinal barrier integrity, while controlling MC activation during malaria. Despite increased intestinal permeability, bacterial 16S levels in blood as a measure of bacteremia in infected basophil-depleted and baso IL-18R were comparable to those of infected controls. We proposed that bacterial translocation was likely controlled by enhanced local and systemic immune responses in mutant mice compared with infected controls (15, 16). Notably, parasite transmission to the mosquito host *Anopheles stephensi* was enhanced from basophil-depleted mice relative to controls, whereas the opposite was true for baso IL-18R (−) mice in that transmission was reduced relative to controls (15, 16).

Given that basophils generate IL-4, IL-13, and histamine in response to IL-18 (17), and that IL-4, IL-13, and IL-18 are increased in plasma in our model (9), we used mice with basophils floxed for the adjacent genes *Il4* and *Il13* [baso IL-4/IL-13 (−) mice] and genotype controls to test the hypothesis that basophil-derived IL-4 and IL-13 control mastocytosis and malaria-induced intestinal permeability and bacteremia. Our data revealed that infected baso IL-4/IL-13 (−) mice exhibited increased ileal mastocytosis, intestinal permeability, and bacteremia relative to infected controls, suggesting that basophil-derived IL-4 and IL-13 blunt MC-dependent intestinal permeability and bacteremia in malaria. We also examined parasite transmission to *A. stephensi* from baso IL-4/IL-13 (−) mice and genotype controls. Although basophil activation regulates parasite transmission to *A. stephensi* (15, 16), our data revealed that basophil-derived IL-4 and IL-13 do not contribute to basophil-dependent control of parasite transmission.

## Materials and Methods

### Mouse strains

Male and female 7- to 8-wk-old mice with basophils deficient for IL-4 and IL-13 [baso IL-4/IL-13 (−)] were generated by crossing basophil-targeted mcpt8-Cre mice (B6.129S4-*Mcpt8^tm1(cre)Lky^*/J; The Jackson Laboratory, 017578) with *Il4/Il13*^flox/flox^ (C.129P2(Cg)-*Il4/Il13^tm1.1Lky^*/J; The Jackson Laboratory, 015859, cryorecovery BALB/c) after breeding onto the C57BL/6J background (18). Littermate Cre (−) *Il4/Il13*^flox/flox^ (baso IL-4/IL-13 (+)) mice were used as controls. Mice were housed in ventilated microisolator cages and provided food and water ad libitum. Basophil-specific knockdown of IL-4 and IL-13 was confirmed with flow cytometry (Supplemental Fig. 1). All procedures were approved by the Institutional Animal Care and Use Committee of the University of Idaho (IACUC protocol 2020-10, approved on March 30, 2020, and renewed as IACUC-2023-08 on February 27, 2023).

### Mouse infection and monitoring

A total of 120 mice (60 females and 60 males) were used across two replicates and injected i.p. with 150 μl of *P. y. yoelii* 17XNL–infected RBCs (1 × 10^6^ parasites) or uninfected RBCs as controls as previously described (9). A total of 100 mice in two replicates were used to assess intestinal permeability and bacteremia, and these were divided into groups for each study time point. A total of 10 mice per time point per replicate were euthanized on days 0, 4, 6, 8, or 10 PI. Daily parasitemias were recorded by microscopic examination of Giemsa-stained thin blood films. Blood, plasma, and ileum samples were collected at euthanasia and processed as described below. A total of 20 baso IL-4/IL-13 (−) and baso IL-4/IL-13 (+) mice (10 males and 10 females of each genotype) were used in two replicates to assess parasite transmission success to *A. stephensi* on day 3 following mouse infection.

### Ileum histochemistry and intestinal permeability

Individual mouse ileum samples (*n* = 100) were formalin fixed and embedded in paraffin (IHC World) for detection of MC chymase using naphthol AS-D chloroacetate esterase (NASDCE) activity (Sigma-Aldrich) according to the manufacturer’s instructions. For assays of intestinal permeability, mice (*n* = 100) were fasted for 4 h, then orally gavaged with 4-kDa FITC-dextran. Blood samples were collected into EDTA 3 h later. Sample fluorescence was quantified with a microplate reader (Molecular Devices, San Jose, CA) at excitation/emission wavelengths of 490/520 nm and compared with a standard curve of serially diluted FITC-dextran in normal mouse plasma with PBS (1:2 v/v) as described (9).

### Bacterial 16S quantitative PCR and ELISAs

Bacterial 16S copy numbers were determined by quantitative PCR (qPCR) in blood samples (*n* = 100) from infected and uninfected mice of both genotypes as described (9). Briefly, whole blood collected in EDTA on the day of necropsy was used to isolate DNA using a DNeasy blood and tissue kit (Qiagen) following the manufacturer’s protocol. Samples were then analyzed in triplicate using SYBR Green/ROX qPCR master mix (2×) (Bio-Rad Laboratories) with 16S primers (9) and quantified against a 16S bacterial DNA plasmid standard curve using a QuantStudio 6 Flex (Applied Biosystems) as described (9). Plasma levels of Mcpt1 (eBioscience), Mcpt4 (eBioscience), neutrophil elastase (NE) (Abcam), myeloperoxidase (MPO) (Abcam), and IgE (eBioscience) were quantified in individual mice (*n* = 100) from the intestinal permeability and bacteremia studies using commercial ELISAs and a microplate reader (Molecular Devices).

### Cytokines and chemokines in plasma and ileum samples

Concentrations of plasma and ileum cytokines and chemokines (IL-1α, IL-1β, IL-2, IL-3, IL-4, IL-5, IL-6, IL-9, IL-10, IL-12p40, IL-12p70, IL-13, IL-17, IFN-γ, TNF-α, MCP-1 [CCL2], MIP-1α [CCL3], MIP-1β [CCL4], RANTES [CCL5], eotaxin [CCL11], GM-CSF, KC [CXCL1]) were quantified in individual mice (*n* = 100) from the intestinal permeability and bacteremia studies using a Bio-Plex Pro Luminex assay on a Bio-Plex 200 system (Bio-Rad Laboratories) and Bio-Plex Manager software (Bio-Rad Laboratories) as described (9, 19).

### Parasite transmission studies

*A. stephensi* Liston was reared and fed on *P. y. yoelii* 17XNL–infected mice as previously described (20). Briefly, baso IL-4/IL-13 (−) mice (*n* = 5 males, 5 females) and baso IL-4/IL-13 (+) mice (*n* = 5 males, 5 females) were infected i.p. with 150 μl of *P. y. yoelii* 17XNL–infected RBCs (1 × 10^6^ parasites) (19) in two replicate studies. Parasitemia, gametocytemia, and exflagellation were assessed at 3 d PI, and each mouse was used to infect 3- to 5-d-old female *A. stephensi* (60 mosquitoes/group). At 10 d postfeeding, 30 fed mosquitoes per group were dissected and microscopically examined (×20 magnification) to count midgut oocysts.

### Statistical analyses

Data from each time point correspond to different mice. Parasitemia, MCs per high-power field, plasma FITC-dextran, bacterial 16S DNA copies per microliter of blood, plasma Mcpt1 and Mcpt4, plasma NE and MPO, as well as ileum and plasma cytokines and chemokines, were analyzed using Brown–Forsythe and Welch ANOVA tests followed by a Dunnett multiple comparison test between baso IL-4/IL-13 (−) and baso IL-4/IL-13 (+) mice at specific time points, and relative to uninfected mice within genotypes. Percentages of infected mosquitoes following feeding on baso IL-4/IL-13 (−) or baso IL-4/IL-13 (+) mice were compared using a Fisher exact test. Numbers of oocysts per midgut from mosquitoes fed on baso IL-4/IL-13 (−) and baso IL-4/IL-13 (+) infected mice, as well as parasitemia and gametocytemia for baso IL-4/IL-13 (−) and baso IL-4/IL-13 (+) mice on day 3 PI, were analyzed using a Kruskal–Wallis. A *p* value ≤0.05 was considered significant. Correlations among parasitemia, plasma FITC-dextran, blood bacterial 16S DNA copies, MC numbers, Mcpt1, Mcpt4, NE, and MPO, as well as ileum and plasma cytokines and chemokines, were assessed by a Spearman test at days 4, 6, 8, and 10 PI. Network analyses were completed with Cytoscape software version 3.10.0 (http://www.cytoscape.org) using significant Spearman correlation coefficients (*p* ≤ 0.05) and immune factors with a fold change >1.5 relative to uninfected controls were included in the network visualizations.

## Results

### Basophil IL-4 and IL-13 deficiency was associated with increased ileal mastocytosis and MC activation

**FIGURE 1.**
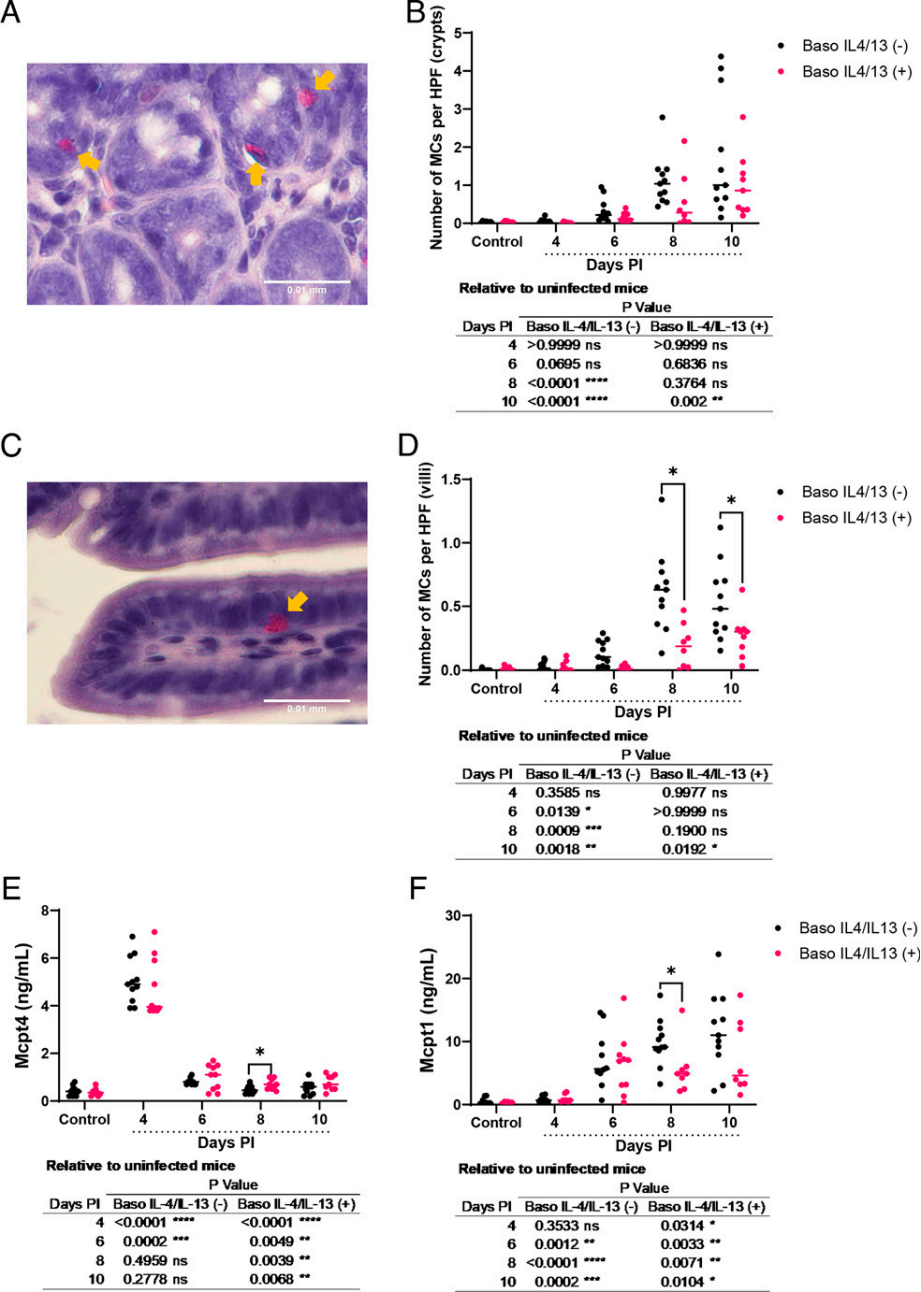
*Plasmodium y. yoelii* 17XNL– infected baso IL-4/IL-13 (−) mice exhibited earlier infection-associated increases in ileal MCs relative to infected baso IL-4/IL-13 (+) mice. (**A**) Representative stained MCs (pink cells indicated by yellow arrows) from naphthol AS-D chloroacetate esterase (NASDCE)–stained sections in the ileum crypts of an infected mouse. (**B**) Numbers of ileal MCs per high-power field (HPF) in ileal crypts from NASDCE-stained sections from uninfected control mice and infected baso IL-4/IL-13 (−) and baso IL-4/IL-13 (+) mice. (**C**) Representative stained MCs (pink cells indicated by yellow arrows) from NASDCE-stained sections in the ileal villi of an infected mouse. (**D**) Numbers of ileal MCs per HPF in ileal villi from NASDCE-stained sections from uninfected control mice and infected baso IL-4/IL-13 (−) and baso IL-4/IL-13 (+) mice. (**E**) Plasma MC protease 4 (Mcpt4) concentrations as determined by ELISA. (**F**) Plasma MC Mcpt1 concentrations as determined by ELISA. Data were analyzed using Brown–Forsythe and Welch ANOVA tests followed by a Dunnett multiple comparison test between infected mice at each time point and uninfected controls, or between baso IL-4/IL-13 (−) and baso IL-4/IL-13 (+) mice at each time point. Each dot represents a single mouse; bars correspond to the mean. A *p* value ≤0.05 was considered significant. *p ≤ 0.05, **p ≤ 0.01, ***p ≤ 0.001, ****p ≤ 0.0001. ns, not significant.

We previously experimentally linked intestinal MC activation to malaria-induced bacteremia (7, 8). Intestinal MC recruitment and activation in mice during both gastrointestinal helminth infection and malaria are marked by MC responsiveness to IL-4, with activated MCs relocating to the villus and returning to the crypts after infection resolution (21–23). Basophils are major sources of IL-4 and IL-13 (24), which are elevated in plasma in our malaria model (19). Given that these cytokines activate MCs (25), we sought to assess how elimination of their synthesis by basophils in malaria affected the development of intestinal mastocytosis in ileum, where immune cells control bacteria (26). In ileal crypts (Fig. 1A), MC numbers trended higher in baso IL-4/IL-13 (−) mice at 6 d PI and were significantly increased relative to uninfected controls by days 8 and 10 PI (Fig. 1B, table). In baso IL-4/IL-13 (+) mice, MCs in crypts were significantly higher relative to controls only at 10 d PI (Fig. 1B, table). There were no differences in MC numbers in crypts between baso IL-4/IL-13 (−) and baso IL-4/IL-13 (+) mice at any time point (Fig. 1B). Similarly, in the ileal villi (Fig. 1C), MC numbers were increased in baso IL-4/IL-13 (−) mice relative to uninfected controls at days 6, 8, and 10 PI (Fig. 1D, table), whereas infected baso IL-4/IL-13 (+) mice exhibited significantly increased MCs in villi relative to uninfected controls only at 10 d PI (Fig. 1D, table). Furthermore, MC numbers in the villi were higher in baso IL-4/IL-13 (−) mice relative to baso IL-4/IL-13 (+) mice at both 8 and 10 d PI (Fig. 1D).

We also assessed MC activation markers Mcpt4 and Mcpt1, which are produced by connective tissue and mucosal MCs, respectively (27, 28), and which have been associated with increased intestinal permeability (19, 29–32). Plasma levels of Mcpt4 peaked at day 4 PI in both baso IL-4/IL-13 (−) and baso IL-4/IL-13 (+) mice (Fig. 1E). Mcpt4 was increased in baso IL-4/IL-13 (−) mice relative to uninfected controls by days 4 and 6 PI (Fig. 1E, table) and decreased relative to baso IL-4/IL-13 (+) mice at day 8 PI (Fig. 1E). In baso IL-4/IL-13 (+) mice, Mcpt4 was increased at days 4, 6, 8, and 10 PI relative to uninfected controls (Fig. 1E, table). Plasma Mcpt1 levels, which were substantially higher than Mcpt4 levels, were increased in baso IL-4/IL-13 (−) mice at days 6, 8, and 10 PI relative to uninfected controls (Fig. 1F, table) and on day 8 PI relative to baso IL-4/IL-13 (+) mice (Fig. 1F). In baso IL-4/IL-13 (+) mice, Mcpt1 levels were increased at days 4, 6, 8, and 10 PI relative to uninfected control mice (Fig. 1F, table). Collectively, these data suggest that basophil-derived IL-4 and IL-13 control the activation and proliferation of MCs after *P. y. yoelii* 17XNL infection.

### Baso IL-4/IL-13 (−) mice showed increased plasma FITC-dextran and bacterial 16S in blood

**FIGURE 2.**
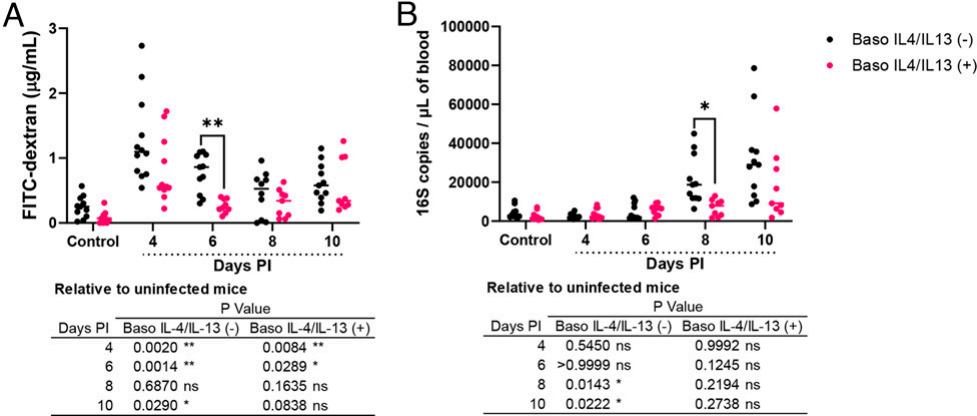
Intestinal permeability and blood bacterial 16S DNA copies/µl were increased in baso IL-4/IL-13 (−) mice during *P. y. yoelii* 17XNL infection. (**A**) Intestinal permeability estimated as plasma FITC-dextran levels after oral gavage and (**B**) bacterial 16S DNA copies/µl of blood as determined by qPCR. Data were analyzed using Brown–Forsythe and Welch ANOVA tests followed by a Dunnett multiple comparisons test between infected mice at each time point and uninfected controls, or between baso IL-4/IL-13 (−) and baso IL-4/IL-13 (+) mice at each time point. Each dot represents a single mouse; bars correspond to the mean. A *p* value ≤0.05 was considered significant. *p ≤ 0.05, **p ≤ 0.01. ns, not significant.

Given our observations of increased ileal mastocytosis and MC activation in baso IL-4/IL-13 (−) mice (Fig. 1), along with our prior observations of MC-dependent intestinal permeability and bacterial translocation in *P. yoelii* infection (7, 8), we sought to assess the effects of basophil-derived IL-4 and IL-13 on intestinal permeability and bacteremia in *P. y. yoelii* 17XNL–infected mice. Plasma levels of FITC-dextran were increased in both baso IL-4/IL-13 (−) and baso IL-4/IL-13 (+) mice at days 4 and 6 PI, and on day 10 PI in baso IL-4/IL-13 (−) mice relative to uninfected controls (Fig. 2A, table). Within time points, FITC-dextran levels trended higher in baso IL-4/IL-13 (−) mice relative to baso IL-4/IL-13 (+) mice at days 4 and 8 PI and were significantly higher at day 6 PI (Fig. 2A). Blood bacterial 16S levels were increased in baso IL-4/IL-13 (−) mice at days 8 and 10 PI relative to uninfected controls (Fig. 2B, table) and were significantly higher and trended higher relative to baso IL-4/IL-13 (+) mice at 8 and 10 d PI, respectively (Fig. 2B). In contrast to baso IL-4/IL-13 (−) mice, blood bacterial 16S levels were not significantly increased in baso IL-4/IL-13 (+) mice relative to uninfected controls at any time point (Fig. 2B, table), suggesting that basophil-derived IL-4 and IL-13 contribute to protection against intestinal permeability and bacterial translocation in our model.

### Basophil-derived IL-4 and IL-13 had no effect on parasitemia

**FIGURE 3.**
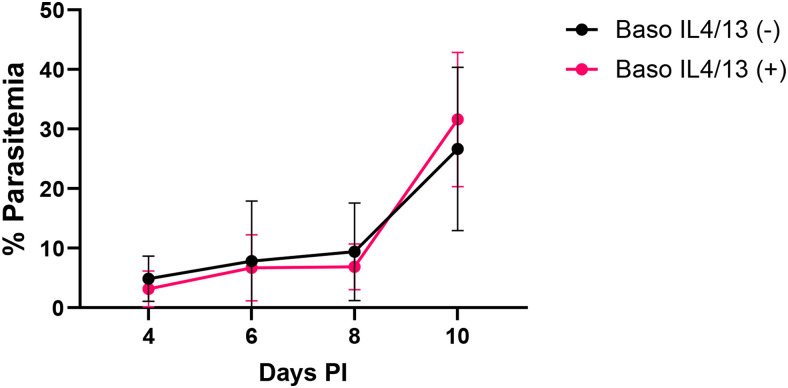
*P. y. yoelii* 17XNL parasitemias were not different between baso IL-4/IL-13 (−) and baso IL-4/IL-13 (+) mice at any time point. Data are represented as means ± SD and were analyzed using Brown–Forsythe and Welch ANOVA tests followed by multiple comparisons between baso IL-4/IL-13 (−) and baso IL-4/IL-13 (+) mice at each time point. A *p* value ≤0.05 was considered significant.

All animals injected with *P. y. yoelii* 17XNL exhibited increased parasitemia with the course of infection (Fig. 3). However, consistent with our previous studies with basophil-depleted mice (15) and mice with basophils lacking IL-18R (16), there were no differences in parasitemia between baso IL-4/IL-13 (−) mice and baso IL-4/IL-13 (+) mice at any time point (Fig. 3). These data affirmed that differences in ileal mastocytosis, MC activation, intestinal permeability, and bacteremia were not confounded by differences in parasite load.

### Neutrophil activation was not significantly affected by the depletion of IL-4 and IL-13 from basophils

**FIGURE 4.**
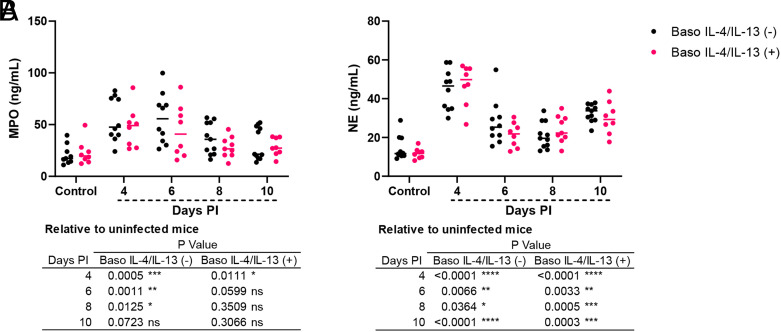
Neutrophil myeloperoxidase levels were increased in baso IL-4/IL-13 (−) mice following *P. y. yoelii* 17XNL infection. (**A**) Plasma myeloperoxidase (MPO) detection by ELISA. (**B**) Plasma neutrophil elastase (NE) as determined by ELISA. Data were analyzed using Brown–Forsythe and Welch ANOVA tests followed by a Dunnett multiple comparisons test between infected mice at each time point and uninfected controls, or between Baso IL4/IL13 (−) and Baso IL4/IL13 (+) mice at each time point. Each dot represents a single mouse; bars correspond to the mean. A *p* value ≤0.05 was considered significant. *p ≤ 0.05, **p ≤ 0.01, ***p ≤ 0.001, ****p ≤ 0.0001. ns, not significant.

Given our observations of increased intestinal permeability and bacteremia in baso IL-4/IL-13 (−) mice (Fig. 2), we assayed plasma MPO and NE (33), antimicrobial effectors frequently used as markers for neutrophil activation (34). Plasma MPO levels were increased at days 4, 6, and 8 PI in baso IL-4/IL-13 (−) mice and at 4 d PI in baso IL-4/IL-13 (+) mice relative to uninfected controls (Fig. 4A, table), but there were no significant differences between genotypes within any time point (Fig. 4A). Plasma NE levels in both baso IL-4/IL-13 (−) and baso IL-4/IL-13 (+) mice were increased at all time points relative to uninfected controls (Fig. 4B, table), but similar to MPO levels, there were no significant differences between genotypes within any time point (Fig. 4B). These data suggest that depletion of IL-4 and IL-13 from basophils did not affect neutrophil activation during infection in our model.

### Network visualization revealed distinct immune response patterns with intestinal permeability and blood bacterial 16S levels in baso IL-4/IL-13 (−) mice relative to baso IL-4/IL-13 (+) mice

**FIGURE 5.**
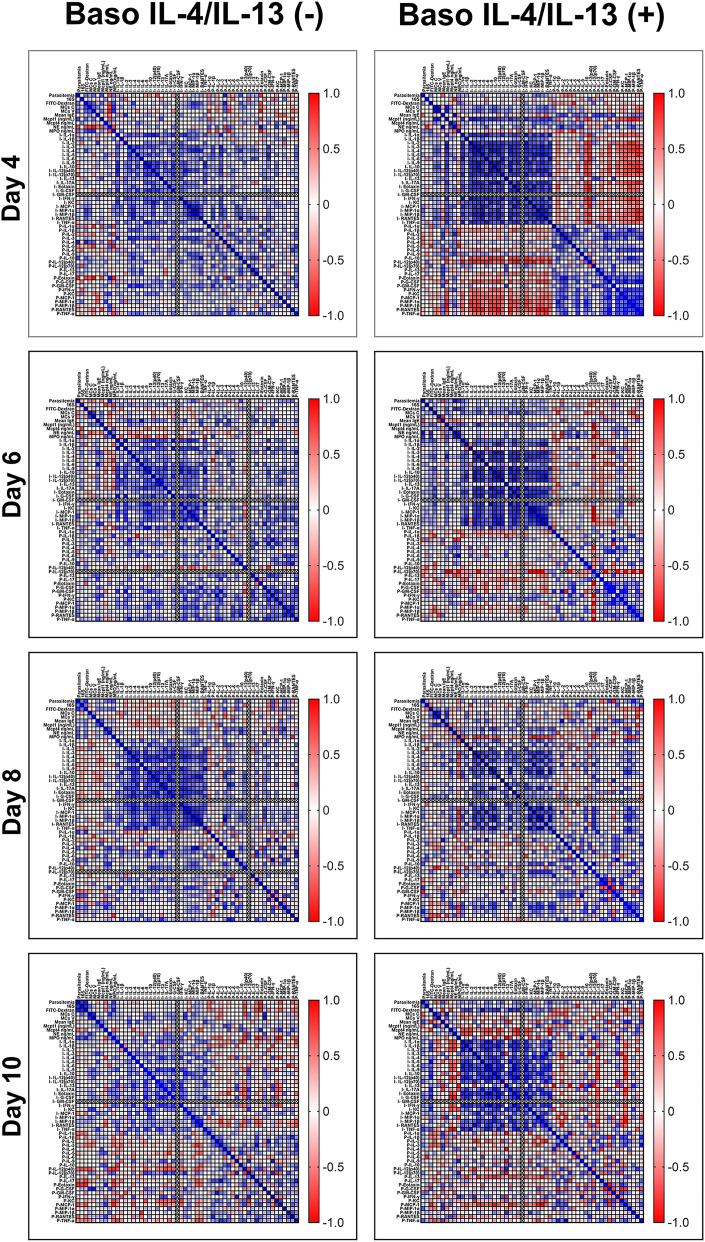
Hierarchical clustering. Heat map representation of Spearman’s correlations between parasitemia, blood bacterial 16S levels, plasma FITC-dextran levels, ileal MC numbers, plasma IgE, Mcpt1, and Mcpt4, and levels of ileal and plasma cytokines and chemokines for *P. y. yoelii* 17XNL–infected baso IL-4/IL-13 (−) mice (left) and baso IL-4/IL-13 (+) mice (right) on days 4, 6, 8, and 10 PI. The heat map colors correspond to correlations grading from 1 (positive correlation, blue), no correlation (white), to −1 (negative correlation, red). A *p* value ≤0.05 was considered significant.

**FIGURE 6.**
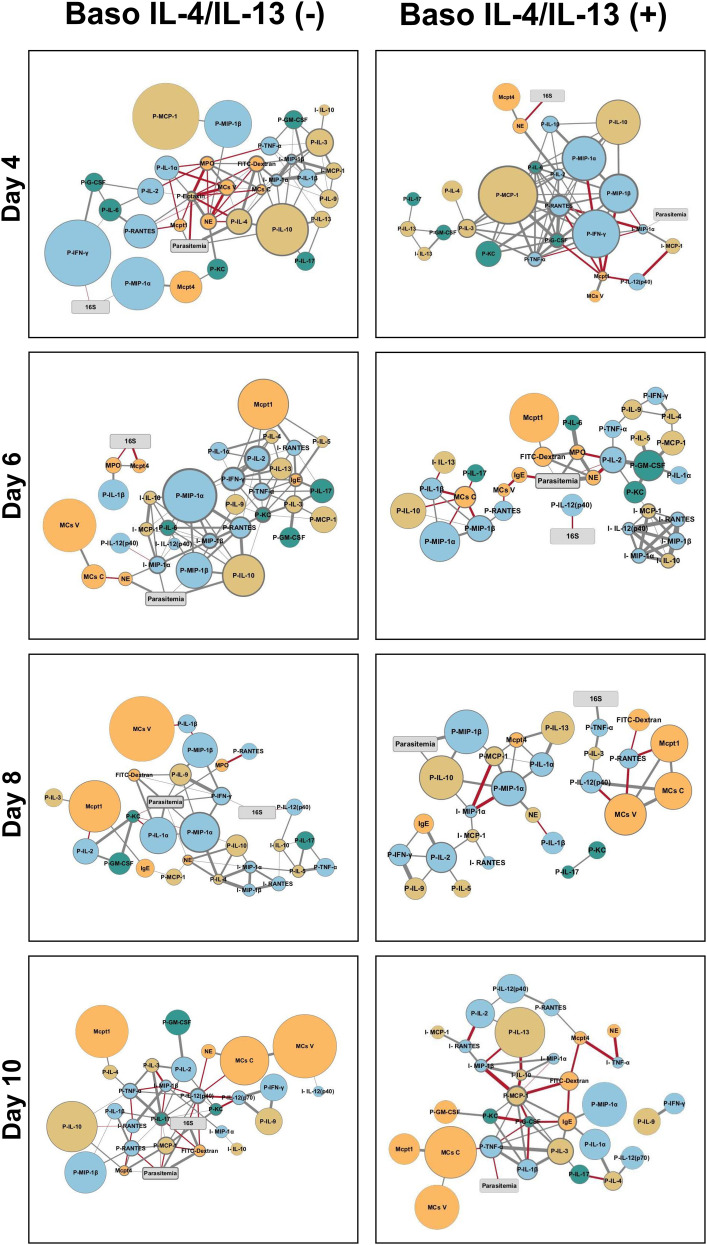
Network visualization of response patterns in baso IL-4/IL-13 (−) and baso IL-4/IL-13 (+) mice during *P. y. yoelii* 17XNL infection. Network visualization of significant correlations (Fig. 5) following infection in baso IL-4/IL-13 (−) (left) and baso IL-4/IL-13 (+) mice (right). The size of a circular node represents relative fold change (larger circle indicates larger fold change) of a parameter calculated as average levels in infected mice/average levels in control, uninfected mice. The border width represents the degree or number of connections (the higher the number of connected edges, the thicker the edge). Parasitemia and bacterial 16S copy nodes are presented in gray, blue nodes are proinflammatory cytokines and chemokines (type 1 immune response), yellow nodes are anti-inflammatory or regulatory cytokines and chemokines (type 2 immune response), green nodes are type 17 immunity-related cytokines and chemokines, and orange nodes represent cells and cell markers (MCs, Mcpt1, Mcpt4, MPO, and NE). Gray strokes connect nodes with positive correlations, and red strokes reflect negative correlations. Increasing stroke line width reflects increasing Spearman correlation value.

We previously observed significant increases in ileal TNF-α and IL-13 as well as profound differences by network analyses of other host immune factors over time during *P. y. yoelii* 17XNL infection in basophil-depleted relative to nondepleted mice (15). In baso IL-18R (−) mice, intestinal permeability was significantly higher relative to baso IL-18R (+) mice at a single time point (4 d PI) that was also marked by significantly higher levels of several proinflammatory cytokines in baso IL-18R (−) mice (16). Given this evident regulation of both malarial pathology and the host immune response by basophils, we evaluated plasma and ileum levels of cytokines and chemokines in baso IL-4/IL-13 (−) mice and genotype controls using correlation and network analyses with patterns of intestinal permeability and blood bacterial 16S levels. In general, there were no significant differences in ileal or plasma levels of cytokines and chemokine between baso IL-4/IL-13 (−) and baso IL-4/IL-13 (+) mice at any time PI (Supplemental Figs. 2, 3). In contrast, however, we observed complex patterns with trends toward increased plasma cytokine levels in baso IL-4/IL-13 (−) mice relative to baso IL-4/IL-13 (+) mice (Supplemental Fig. 3). Accordingly, we created correlation matrices for data from baso IL-4/IL-13 (−) mice and baso IL-4/IL-13 (+) mice to identify significant relationships between parasitemia, bacteremia, and intestinal permeability with ileal MCs, plasma and ileal cytokines and chemokines, and plasma IgE, Mcpt1, Mcpt4, MPO, and NE over time (Fig. 5). Significant correlations (*p* < 0.05) with fold increases >1.5 relative to uninfected controls were then used to build interaction networks by day and by group (Fig. 6), facilitating the identification of time- and state-dependent relationships without biases based on known contributors to an outcome.

At days 4, 6, 8, and 10 PI, visually distinct patterns between infection parameters from baso IL-4/IL-13 (−) mice and baso IL-4/IL-13 (+) mice (Fig. 5) provided ample significant correlations and fold differences for network analyses. At 4 d PI in baso IL-4/IL-13 (−) mice, plasma eotaxin (CCL11), well known for its roles in allergic inflammation (35–37), had the highest number of connected nodes (*n* = 11). Eotaxin was positively correlated with NE and plasma RANTES (CCL5), IL-1α, and IL-2, and strongly negatively correlated with MPO, Mcpt1, parasitemia, plasma IL-4, ileal MCs, and FITC-dextran. Moreover, FITC-dextran levels were negatively correlated with NE, and positively correlated with MPO, ileum chemokines MIP-1α (CCL3) and MIP-1β (CCL4), and plasma cytokines IL-4 and IL-10, as well as MCs in crypts and villi. These patterns could, in part, reflect the fact that blockade of IL-4/IL-13 can result in inhibition of eotaxin and lack of migration of eosinophils into tissues (38). Blood bacterial 16S copies were negatively correlated with plasma IFN-γ and positively correlated with plasma MIP-1α (CCL3) (Fig. 6). In baso IL-4/IL-13 (+) mice, FITC-dextran levels were not correlated with any of the markers measured, and blood bacterial 16S copies were correlated only with NE and this was a negative correlation (Fig. 6).

At 6 d PI in baso IL-4/IL-13 (−) mice, FITC-dextran levels were not correlated with any of the markers measured. Bacterial 16S copies were negatively correlated with MPO and Mcpt4 outside of the core network, which was mainly driven by parasitemia (Fig. 6). In baso IL-4/IL-13 (+) mice, FITC-dextran levels were positively correlated with MPO, NE, Mcpt1, and parasitemia in the main network, and bacterial 16S copies were negatively correlated with plasma IL-12p40, which was isolated from the main network (Fig. 6).

At 8 d PI in baso IL-4/IL-13 (−) mice, FITC-dextran levels were positively correlated with parasitemia, plasma IL-1α, IFN-γ and IL-9, while bacterial 16S copies were positively correlated with plasma IFN-γ and IL-12p40 (Fig. 6). In baso IL-4/IL-13 (+) mice, FITC-dextran levels were negatively correlated with plasma RANTES (CCL5), and together with 16S copies, which were positively correlated with TNF-α, they formed a separate network driven by MCs in crypts and villi as well as the MC marker Mcpt1 (Fig. 6).

At 10 d PI in baso IL-4/IL-13 (−) mice, FITC-dextran levels were positively correlated with bacterial 16S copies and parasitemia and negatively correlated with plasma IL-12p40, MCP-1, and IL-17, with the latter being the center of a larger network of positively correlated type 1 plasma cytokines TNF-α, IL-12p40, IL-1β, and RANTES and negatively correlated ileal MIP-1β (Fig. 6). Bacterial 16S copies, which were networked similarly to FITC-dextran, were positively correlated with parasitemia, and negatively correlated with plasma IL-12p40. In baso IL-4/IL-13 (+) mice, FITC-dextran levels were positively correlated with plasma IL-10 and negatively correlated to a plasma MCP-1 (CCL2)–centered network, IgE, and Mcpt4, whereas bacterial 16S copies were absent from the network. Parasitemia was negatively correlated with plasma TNF-α, which was also positively correlated with an MCP-1–centered network (Fig. 6).

### Basophil derived IL-4 and IL-13 had no effect on transmission of *P. yoelii* to *A. stephensi*

**FIGURE 7.**
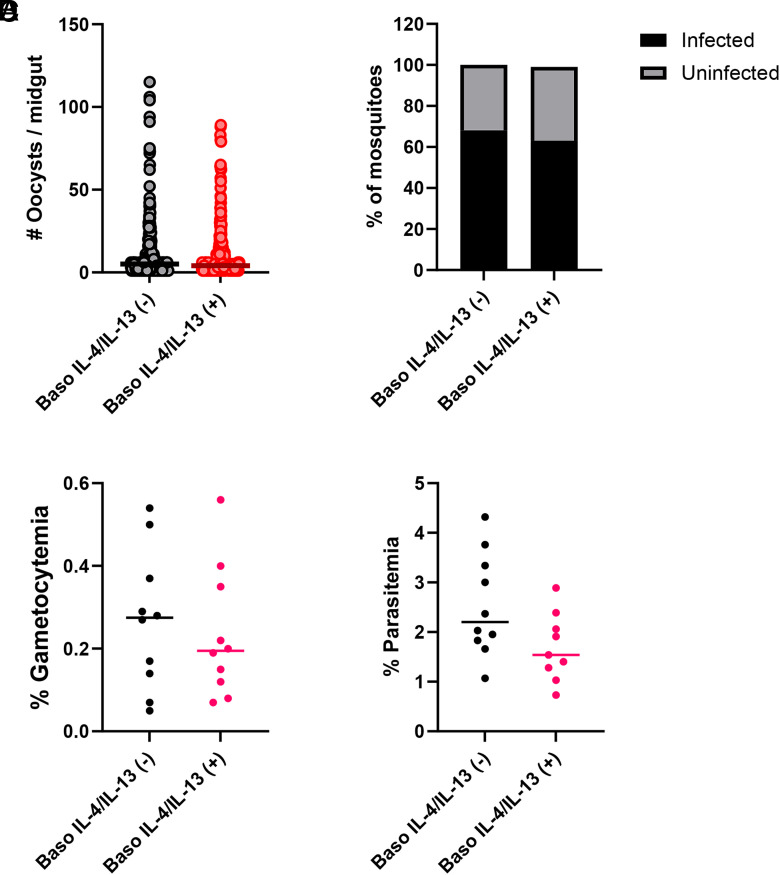
Transmission of *P. y. yoelii* 17XNL to *A. stephensi*. (**A**) Numbers of oocysts per mosquito midgut. (**B**) Proportions of mosquitoes infected with oocysts. (**C** and **D**) Percentages of mouse erythrocytes infected with sexual stage gametocytes (C) and asexual stages (D) on the day of mosquito infection (day 3 PI). Data from oocyst, parasitemia, and gametocytemia were analyzed by a Mann–Whitney *U* test. The percentages of infected mosquitoes (mosquitoes with no oocysts excluded) were analyzed with a Fisher exact test.

In our model, basophils are significantly increased in circulation by day 4 PI (19), a time that closely follows maximal transmission of *P. yoelii* to *A. stephensi* at 3–4 d PI. Subsequently, we reported that mice depleted of basophils exhibited increased gametocytemia, and mosquitoes that fed on these mice developed greater numbers of parasites relative to infected, nondepleted mice (15). On the contrary, in mice with basophils depleted of IL-18R, gametocyte numbers were not significantly different between genotypes, but the percentage of infected mosquitoes was lower in mosquitoes that fed on baso IL-18R (−) mice relative to those that fed on baso IL-18R (+) mice (16). Based on these observations, we sought to test the effects of basophil-derived IL-4 and IL-13 on gametocytemia and parasite transmission. Mean oocysts per mosquito midgut (Fig. 7A) and percentages of infected mosquitoes (Fig. 7B) following feeding on infected baso IL-4/IL-13 (−) mice relative to infected baso IL-4/IL-13 (+) mice were not significantly different. These observations were consistent with nonsignificant trends toward higher gametocytemia (Fig. 7C) and parasitemia (Fig. 7D) in baso IL-4/IL-13 (−) mice relative to baso IL-4/IL-13 (+) mice. Accordingly, depletion of basophil-derived IL-4 and IL-13 had little to no effect on gametocytemia and no effect on parasite transmission to *A. stephensi*.

## Discussion

Under normal health conditions, basophils are the least abundant circulating granulocytes (39). Upon activation, these cells degranulate to release histamine as well as cytokines and chemokines, including inducible IL-4 and IL-13 (40, 41). Given the rarity of basophils and the fact that other cell types produce IL-4 and IL-13, however, we did not expect to see differences in IL-4 and IL-13 levels between baso IL-4/IL-13 (−) mice and baso IL-4/IL-13 (+) mice. Reports indicate that IL-18 stimulates NKT cells, which are the primary source of IL-4 (42, 43). Type 2 innate lymphoid cells are also a major source of IL-13 (44), and both can serve as important sources of IL-4 and mediators of the Th2 immune response in a tissue-specific manner (45), as well as in the context of *Plasmodium chabaudi* AS infection (46), suggesting that our focus on basophil-derived IL-4 and IL-13 can help to bring greater understanding of basophil-specific effects in malaria.

IL-4 and IL-13, in addition to cross-linking FcεRI and activating TLR signaling, can activate MCs, which are major regulators of the intestinal barrier (47–49). Activated basophils can contribute to both tissue pathology and tissue repair. For example, basophilia that is independent of MCs has been associated with inflammatory bowel diseases (50, 51), but in the context of myocardial infarction, basophils have been associated with tissue repair by increasing IL-4 and IL-13 levels, and altering local monocyte/macrophage responses (52). In the present studies, we observed a trend toward increased intestinal permeability in baso IL-4/IL-13 (−) mice at day 4 PI, a pattern that was significant at day 6 PI (Fig. 2A). We previously reported increased intestinal permeability relative to controls in mice with basophils lacking IL-18R at day 4 PI (16) and in basophil-depleted mice at days 4, 6, and 8 PI (15). We also observed increased bacterial 16S copies in baso IL-4/IL-13 (−) mice relative to baso IL-4/IL-13 (+) mice at 8 d PI with a similar trend at 10 d PI (Fig. 2B), a phenotype that was not observed in our previous models (15, 16). These observations confirm a protective role of basophils and suggest that basophil-derived IL-4 and IL-13 protect against *P. y. yoelii*–induced intestinal barrier damage and, therefore, the translocation of enteric bacteria across the damaged barrier.

On day 4 PI, FITC-dextran was significantly negatively correlated with plasma eotaxin (CCL11), a potent eosinophil chemoattractant (53) that plays a major role in allergic inflammation (35–37). Eosinophils are frequently associated with chronic inflammatory conditions of the gastrointestinal tract and eosinophil-associated gastrointestinal disorders (54). In addition to eotaxin, FITC-dextran was positively correlated with MPO, ileum chemokines MIP-1α (CCL3) and MIP-1β (CCL4), plasma cytokines IL-4 and IL-10, as well as ileal MCs (Fig. 6), indicative of a strong inflammatory response.

Increased levels of FITC-dextran were also associated with increased MCs in ileal crypts (Fig. 1B) and villi (Fig. 1D) in baso IL-4/IL-13 (−) mice relative to uninfected controls, with significant differences in the villi relative to baso IL-4/IL-13 (+) mice at days 8 and 10 PI (Fig. 1D). Previous studies have linked intestinal helminth infections in mice to the movement of MCs from the submucosa to the villus tips, where they return once the infection and inflammation have resolved (23). The observed differences in MC localization between baso IL-4/IL-13 (−) mice and baso IL-4/IL-13 (+) mice suggest that MCs are functionally linked to alterations in gut homeostasis in this model. Moreover, the peak of villi MCs at day 8 PI was associated with significantly increased plasma Mcpt1 and decreased Mcpt4 in baso IL-4/IL-13 (−) mice relative to baso IL-4/IL-13 (+) mice (Fig. 1F). We previously observed increased intestinal permeability, altered ileal adherens junction E-cadherin, and ileal MC accumulation in Mcpt4 knockout mice relative to wild-type mice (19). Although the underlying mechanisms are unknown, these results, together with our observation of reduced Mcpt4 expression in baso IL-4/IL-13 (−) mice compared with control mice, suggest that differential activation of MCs may play a role in maintaining gut homeostasis in this model. In addition, increased intestinal permeability in our previous models relative to genotype controls following infection was also associated with increased intestinal MCs, specifically in basophil-depleted mice by 8 d PI (15) and in mice with basophils depleted of IL-18R by 10 d PI (16). Mucosal MCs require IL-3 or stem cell factor for their proliferation, but IL-4 from basophils or other cell sources including IL-33–induced type 2 innate lymphoid cells or T cells can enhance and directly stimulate the effects of these cytokines (55, 56). Altogether, these data suggest that basophil-derived IL-4 regulates MC activation and proliferation in the context of disruption of barrier integrity in malaria as previously described (7, 29, 30, 32, 47).

Later after infection (day 10), FITC-dextran levels in baso IL-4/IL-13 (−) mice were negatively correlated with IL-17A, which was the center of a larger network of positively correlated type 1 plasma cytokines (Fig. 6). In a model of kidney fibrosis, it has been suggested that basophil-derived IL-6 contributed to enhanced Th17 cell differentiation from CD4^+^ T cells, which contribute to renal fibrosis (57). Moreover, in a model of inflammatory bowel disorder, basophils, when activated by IL-3 and IL-33, can increase IL-17 production and promote the differentiation of Th17 cells, thus contributing to pathogenesis (50).

In addition to increased intestinal permeability, we observed increased bacterial 16S copies in blood of baso IL-4/IL-13 (−) mice relative to genotype controls following infection (Fig. 2B). Enhanced intestinal permeability was previously observed in infected basophil-depleted and baso IL-18R(−) mice, but this was not associated with increased bacterial 16S copies in blood relative to genotype controls (15, 16). This could be explained by differences in the immune response in these mutant mice. Specifically, basophil-depleted mice exhibited significantly increased ileal levels of the proinflammatory cytokine TNF-α (15), but this was not observed in baso IL-4/IL-13 (−) mice. TNF-α is an important contributor to host defense against bacterial infections (58–60). Baso IL-18R(−) mice exhibited cytokine profiles with most cytokines trending lower relative to their genotype controls in ileum, and most cytokines trending higher by day 4 PI in plasma (16). Major differences, however, were noted for the plasma cytokines IL-1β and IL-17A, which were significantly elevated in baso IL-18R(−) mice relative to baso IL-18R(+) mice (16). IL-1β and IL-17A can promote neutrophil recruitment (61) and stimulate the synthesis of inflammatory mediators including G-CSF, KC (CXCL1), TNF-α, and IL-6 (62), cytokines that were also elevated in baso IL-18R(−) mice during infection. At day 8 PI when bacterial 16S copies were significantly higher in baso IL-4/IL-13 (−) mice compared with baso IL-4/IL-13 (+) mice (Fig. 2B), network analyses revealed that 16S copies in baso IL-4/IL-13 (−) mice were positively but weakly correlated with plasma proinflammatory cytokines IFN-γ and IL-12p40 (Fig. 6), whereas in baso IL-4/IL-13 (+) mice, 16S copies were strongly positively correlated with TNF-α, forming a network with FITC-dextran, plasma RANTES (CCL5), and MCs (Fig. 6). There were no differences, however, in plasma levels of the neutrophil markers MPO and NE in infected baso IL-4/IL-13 (−) and baso IL-4/IL-13 (+) mice (Fig. 4). Taken together, these data suggest that mice with basophils deficient for IL-4 and IL-13 failed to control bacterial translocation into the blood during infection and this was not dependent on a lack of neutrophil activation.

We observed no differences in parasite transmission of *P. y. yoelii* 17XNL to *A. stephensi* following mosquito feeding on infected baso IL-4/IL-13 (−) mice versus baso IL-4/IL-13 (+) mice (Fig. 7). In comparison, infection levels of mosquitoes fed on infected basophil-depleted mice were higher than genotype controls (15), whereas infection levels of mosquitoes fed on infected baso IL-18R (−) mice were lower than those of genotype controls (16). These differences in transmission could be explained by the differences in the early immune response to *P. yoelii* in our models. Early after infection (day 4 PI), plasma levels of IFN-γ, TNF-α, IL-3, IL-17, IL-1β, and IL-4 were increased in baso IL-18R (−) mice (16) but not in basophil-depleted mice (15). These cytokines are markers of a hyperinflammatory state, with IFN-γ negatively associated with gametocyte commitment and parasite transmission (63). Infected baso IL-4/IL-13 (−) mice exhibited cytokine trends similar to those observed in baso IL-18R (−) mice, but plasma levels in baso IL-4/IL-13 (−) mice were not significantly different from those in baso IL-4/IL-13 (+) mice (Supplemental Fig. 3). Based on these patterns, we speculate that a systemic hyperinflammatory state in baso IL-18R (−) mice, a moderate inflammatory response in baso IL-4/IL-13 (−) mice, and a suppressed inflammatory response in basophil-depleted mice relative to genotype controls could underlie observed patterns of suppressed, equivalent, and enhanced parasite transmission, respectively, to *A. stephensi*.

In summary, we confirmed that basophil activation and the synthesis of Th2 cytokines IL-4 and IL-13 protect intestinal barrier integrity after *P. yoelii* infection, thereby controlling MC activation and proliferation and preventing infection-induced intestinal barrier damage, enteric bacterial translocation into the blood, and therefore protecting from and reducing the risk of sepsis (64, 65). This is possibly orchestrated by controlling eosinophils, macrophages, and Th17-mediated inflammation. Further studies are needed to determine the impact of basophil-derived IL-4 and IL-13 on eosinophil and macrophage function. We have also confirmed that basophil activation controls parasite transmission (15, 16) and have now eliminated basophil-derived IL-4 and IL-13 from among the factors responsible for this regulation. Although we have not fully defined the mechanisms whereby malaria-induced basophil activation controls these phenotypes, between the current dataset and previous studies, we now have a robust set of infection parameters (9, 15, 16) to help refine specific pathways of basophil-dependent control of intestinal barrier integrity and parasite transmission in malaria.

## Supplementary Material

Supplemental Material (PDF)
