# Supplementary material for: Basophil-Derived IL-4 and IL-13 Protect Intestinal Barrier Integrity and Control Bacterial Translocation during Malaria
Source: Immunohorizons. 2024 May 23;8(5):371–83. doi: 10.4049/immunohorizons.2300084 (PMC11150129; doi:10.4049/immunohorizons.2300084)
Supplement: Supplemental Material (PDF) [file IH_2300084_Supplemental_1.pdf]

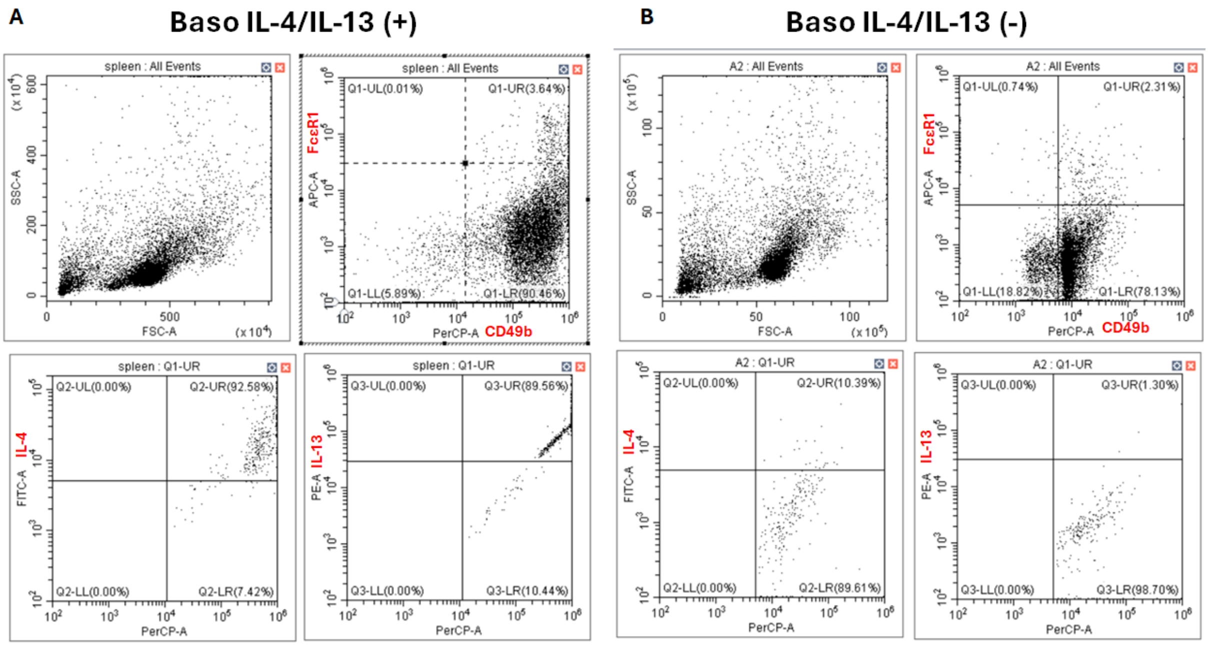

Supplementary Figure 1. Flow cytometry to confirm depletion of IL-4 and IL-13 on mouse basophils. Basophils were collected from spleens of female baso IL-4/IL-13 (+) **(A)** and baso IL-4/IL-13 **(-)** (B) mice at day 4 PI. A total of 10^6 cells were incubated with anti-mouse CD49 (PerCP) (eBioscience) and anti-mouse FcεRI (APC) (eBioscience) at the manufacturer's recommended concentrations, then stained with DAPI (Invitrogen), anti-mouse IL-4 (FITC) (eBioscience) and anti-mouse IL-13 (PE) after cell permeabilization. Stained cells were counted using a CytoFLEX flow cytometer (Beckman Coulter, Brea, CA). Live granulocytes were selected based on forward/side scatter and DAPI staining, and basophils within this population were defined as expressing both CD49 and FCεRI. Basophils expressing intracellular IL-14 and IL-13 were counted and data were analyzed with CytExpert software (Beckman Coulter). In baso IL-4/IL-13 (+) mice, IL-4 was expressed in 92.6% of basophils (panel A, lower left), and IL-13 was expressed in 89.6 % of basophils (panel A, lower right), while in baso IL-4/IL-13 (-) mice, IL-4 was expressed in 10% of basophils (panel B, lower left), and IL-13 was expressed in 1.3 % of basophils (panel B, lower right).

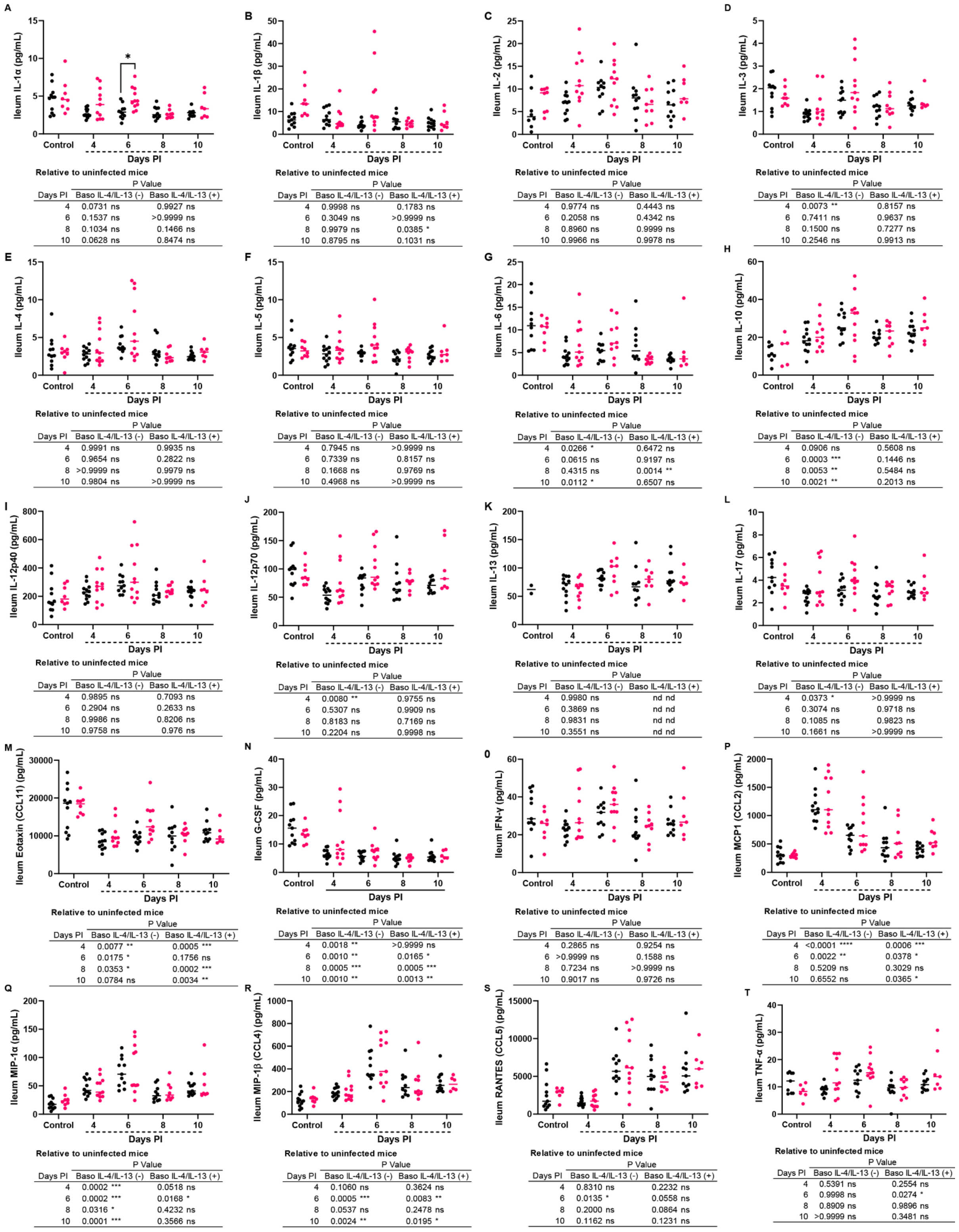

A — Relative to uninfected mice

Days PI	P Value: Baso IL-4/IL-13 (-)	P Value: Baso IL-4/IL-13 (+)
4	0.0731 ns	0.9927 ns
6	0.1537 ns	>0.9999 ns
8	0.1034 ns	0.1466 ns
10	0.0628 ns	0.8474 ns

B — Relative to uninfected mice

Days PI	P Value: Baso IL-4/IL-13 (-)	P Value: Baso IL-4/IL-13 (+)
4	0.9998 ns	0.1783 ns
6	0.3049 ns	>0.9999 ns
8	0.9979 ns	0.0385 *
10	0.8795 ns	0.1031 ns

C — Relative to uninfected mice

Days PI	P Value: Baso IL-4/IL-13 (-)	P Value: Baso IL-4/IL-13 (+)
4	0.9774 ns	0.4443 ns
6	0.2058 ns	0.4342 ns
8	0.8960 ns	0.9999 ns
10	0.9966 ns	0.9978 ns

D — Relative to uninfected mice

Days PI	P Value: Baso IL-4/IL-13 (-)	P Value: Baso IL-4/IL-13 (+)
4	0.0073 **	0.8157 ns
6	0.7411 ns	0.9637 ns
8	0.1500 ns	0.7277 ns
10	0.2546 ns	0.9913 ns

E — Relative to uninfected mice

Days PI	P Value: Baso IL-4/IL-13 (-)	P Value: Baso IL-4/IL-13 (+)
4	0.9991 ns	0.9935 ns
6	0.9654 ns	0.2822 ns
8	>0.9999 ns	0.9979 ns
10	0.9804 ns	>0.9999 ns

F — Relative to uninfected mice

Days PI	P Value: Baso IL-4/IL-13 (-)	P Value: Baso IL-4/IL-13 (+)
4	0.7945 ns	>0.9999 ns
6	0.7339 ns	0.8157 ns
8	0.1668 ns	0.9769 ns
10	0.4968 ns	>0.9999 ns

G — Relative to uninfected mice

Days PI	P Value: Baso IL-4/IL-13 (-)	P Value: Baso IL-4/IL-13 (+)
4	0.0266 *	0.6472 ns
6	0.0615 ns	0.9197 ns
8	0.4315 ns	0.0014 **
10	0.0112 *	0.6507 ns

H — Relative to uninfected mice

Days PI	P Value: Baso IL-4/IL-13 (-)	P Value: Baso IL-4/IL-13 (+)
4	0.0906 ns	0.5608 ns
6	0.0003 ***	0.1446 ns
8	0.0053 **	0.5484 ns
10	0.0021 **	0.2013 ns

I — Relative to uninfected mice

Days PI	P Value: Baso IL-4/IL-13 (-)	P Value: Baso IL-4/IL-13 (+)
4	0.9895 ns	0.7093 ns
6	0.2904 ns	0.2633 ns
8	0.9986 ns	0.8206 ns
10	0.9758 ns	0.976 ns

J — Relative to uninfected mice

Days PI	P Value: Baso IL-4/IL-13 (-)	P Value: Baso IL-4/IL-13 (+)
4	0.0080 **	0.9755 ns
6	0.5307 ns	0.9909 ns
8	0.8183 ns	0.7169 ns
10	0.2204 ns	0.9998 ns

K — Relative to uninfected mice

Days PI	P Value: Baso IL-4/IL-13 (-)	P Value: Baso IL-4/IL-13 (+)
4	0.9980 ns	nd nd
6	0.3869 ns	nd nd
8	0.9831 ns	nd nd
10	0.3551 ns	nd nd

L — Relative to uninfected mice

Days PI	P Value: Baso IL-4/IL-13 (-)	P Value: Baso IL-4/IL-13 (+)
4	0.0373 *	>0.9999 ns
6	0.3074 ns	0.9718 ns
8	0.1085 ns	0.9823 ns
10	0.1661 ns	>0.9999 ns

M — Relative to uninfected mice

Days PI	P Value: Baso IL-4/IL-13 (-)	P Value: Baso IL-4/IL-13 (+)
4	0.0077 **	0.0005 ***
6	0.0175 *	0.1756 ns
8	0.0353 *	0.0002 ***
10	0.0784 ns	0.0034 **

N — Relative to uninfected mice

Days PI	P Value: Baso IL-4/IL-13 (-)	P Value: Baso IL-4/IL-13 (+)
4	0.0018 **	>0.9999 ns
6	0.0010 **	0.0165 *
8	0.0005 ***	0.0005 ***
10	0.0010 **	0.0013 **

0 — Relative to uninfected mice

Days PI	P Value: Baso IL-4/IL-13 (-)	P Value: Baso IL-4/IL-13 (+)
4	0.2865 ns	0.9254 ns
6	>0.9999 ns	0.1588 ns
8	0.7234 ns	>0.9999 ns
10	0.9017 ns	0.9726 ns

P — Relative to uninfected mice

Days PI	P Value: Baso IL-4/IL-13 (-)	P Value: Baso IL-4/IL-13 (+)
4	<0.0001 ****	0.0006 ***
6	0.0022 **	0.0378 *
8	0.5209 ns	0.3029 ns
10	0.6552 ns	0.0365 *

Q — Relative to uninfected mice

Days PI	P Value: Baso IL-4/IL-13 (-)	P Value: Baso IL-4/IL-13 (+)
4	0.0002 ***	0.0518 ns
6	0.0002 ***	0.0168 *
8	0.0316 *	0.4232 ns
10	0.0001 ***	0.3566 ns

R — Relative to uninfected mice

Days PI	P Value: Baso IL-4/IL-13 (-)	P Value: Baso IL-4/IL-13 (+)
4	0.1060 ns	0.3624 ns
6	0.0005 ***	0.0083 **
8	0.0537 ns	0.2478 ns
10	0.0024 **	0.0195 *

S — Relative to uninfected mice

Days PI	P Value: Baso IL-4/IL-13 (-)	P Value: Baso IL-4/IL-13 (+)
4	0.8310 ns	0.2232 ns
6	0.0135 *	0.0558 ns
8	0.2000 ns	0.0864 ns
10	0.1162 ns	0.1231 ns

T — Relative to uninfected mice

Days PI	P Value: Baso IL-4/IL-13 (-)	P Value: Baso IL-4/IL-13 (+)
4	0.5391 ns	0.2554 ns
6	0.9998 ns	0.0274 *
8	0.8909 ns	0.9896 ns
10	>0.9999 ns	0.3481 ns

Supplementary Figure 2. Ileal cytokines and chemokines from *P. y. yoelii* 17XNL-infected Baso IL-4/IL-13 (-) and Baso IL-4/IL-13 (+) mice and uninfected controls. The y axis represents the concentrations. Data were analyzed using Brown-Forsythe and Welch ANOVA tests followed by Dunnett's multiple comparisons test between infected mice at each time point and uninfected controls, or between Baso IL4/IL13 (-) and Baso IL4/IL13 (+) mice at each time point. Each dot represents a single mouse, bars correspond to the mean. P values ≤ 0.05 were considered significant. *P≤0.05, **P≤0.01, ***P≤0.001, ****P≤0.0001, ns: not significant. nd: no determined. Black dots (Baso IL-4/IL-13 (-) and pink dots Baso IL-4/IL-13 (+).

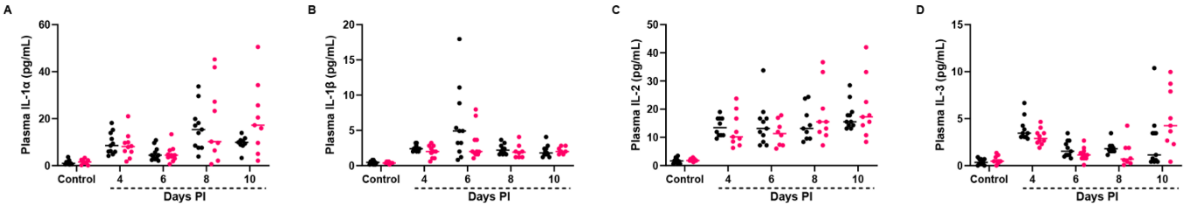

A Relative to uninfected mice

	P Value	
Days PI	Baso IL-4/IL-13 (-)	Baso IL-4/IL-13 (+)
4	0.0019 **	0.0269 *
6	0.0177 *	0.1022 ns
8	0.0048 **	0.144 ns
10	0.0001 ***	0.0623 ns

B Relative to uninfected mice

	P Value	
Days PI	Baso IL-4/IL-13 (-)	Baso IL-4/IL-13 (+)
4	<0.0001 ****	0.0025 **
6	0.0633 ns	0.0328 *
8	0.0001 ***	0.0225 *
10	0.0007 ***	0.0005 ***

C Relative to uninfected mice

	P Value	
Days PI	Baso IL-4/IL-13 (-)	Baso IL-4/IL-13 (+)
4	<0.0001 ****	0.0085 **
6	0.0026 **	0.0059 **
8	0.0021 **	0.0143 *
10	<0.0001 ****	0.0108 *

D Relative to uninfected mice

	P Value	
Days PI	Baso IL-4/IL-13 (-)	Baso IL-4/IL-13 (+)
4	<0.0001 ****	<0.0001 ****
6	0.0036 **	0.2724 ns
8	0.0002 ***	0.9064 ns
10	0.2981 ns	0.0369 *

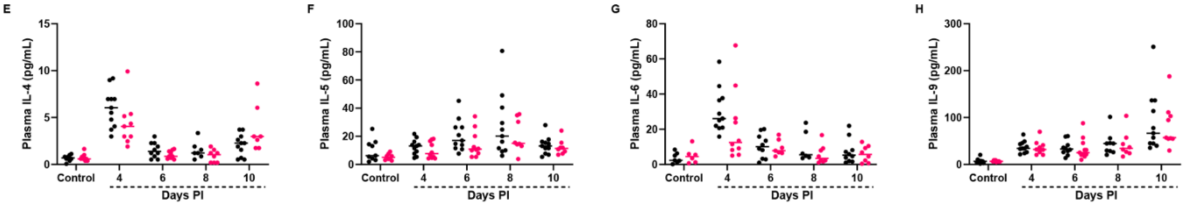

E Relative to uninfected mice

	P Value	
Days PI	Baso IL-4/IL-13 (-)	Baso IL-4/IL-13 (+)
4	<0.0001 ****	0.0214 *
6	0.1035 ns	0.8039 ns
8	0.3843 ns	0.9997 ns
10	0.0297 *	0.1557 ns

F Relative to uninfected mice

	P Value	
Days PI	Baso IL-4/IL-13 (-)	Baso IL-4/IL-13 (+)
4	0.9977 ns	0.3151 ns
6	0.2197 ns	0.1508 ns
8	0.3026 ns	0.0713 ns
10	0.9716 ns	0.0844 ns

G Relative to uninfected mice

	P Value	
Days PI	Baso IL-4/IL-13 (-)	Baso IL-4/IL-13 (+)
4	<0.0001 ****	0.1163 ns
6	0.8041 ns	>0.9999 ns
8	>0.9999 ns	>0.9999 ns
10	>0.9999 ns	>0.9999 ns

H Relative to uninfected mice

	P Value	
Days PI	Baso IL-4/IL-13 (-)	Baso IL-4/IL-13 (+)
4	<0.0001 ****	0.0047 **
6	0.0024 **	0.0295 *
8	0.0168 *	0.0724 ns
10	0.0174 *	0.0137 *

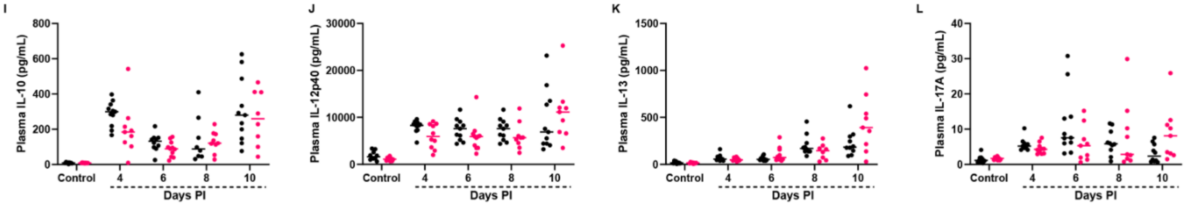

I Relative to uninfected mice

	P Value	
Days PI	Baso IL-4/IL-13 (-)	Baso IL-4/IL-13 (+)
4	<0.0001 ****	0.0452 *
6	0.0005 ***	0.0014 **
8	0.2090 ns	0.0044 **
10	0.0037 **	0.0238 *

J Relative to uninfected mice

	P Value	
Days PI	Baso IL-4/IL-13 (-)	Baso IL-4/IL-13 (+)
4	<0.0001 ****	0.0027 **
6	<0.0001 ****	0.0068 **
8	<0.0001 ****	0.0098 **
10	0.0367 *	0.0133 *

K Relative to uninfected mice

	P Value	
Days PI	Baso IL-4/IL-13 (-)	Baso IL-4/IL-13 (+)
4	0.0079 **	0.0015 **
6	0.0014 **	0.0559 ns
8	0.0052 **	0.0458 *
10	0.0170 *	0.0346 *

L Relative to uninfected mice

	P Value	
Days PI	Baso IL-4/IL-13 (-)	Baso IL-4/IL-13 (+)
4	0.0008 ***	0.002 **
6	0.0482 *	0.3073 ns
8	0.0206 *	0.5311 ns
10	0.5618 ns	0.2028 ns

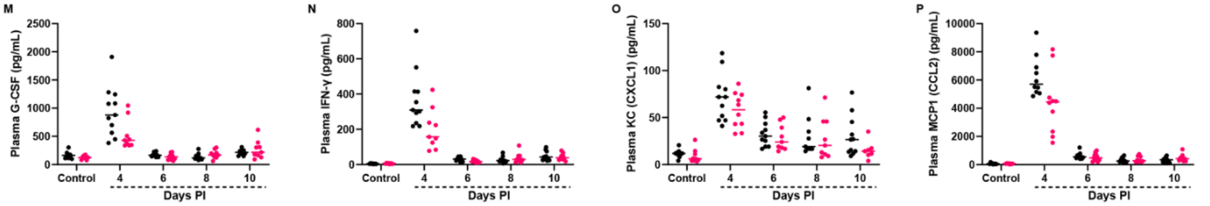

M Relative to uninfected mice

	P Value	
Days PI	Baso IL-4/IL-13 (-)	Baso IL-4/IL-13 (+)
4	0.002 **	0.0204 *
6	>0.9999 ns	0.9993 ns
8	0.9682 ns	0.4918 ns
10	0.4879 ns	0.2613 ns

N Relative to uninfected mice

	P Value	
Days PI	Baso IL-4/IL-13 (-)	Baso IL-4/IL-13 (+)
4	0.0003 ***	0.0102 *
6	0.0007 ***	0.0069 **
8	0.0436 *	0.109 ns
10	0.0033 **	0.0045 **

O Relative to uninfected mice

	P Value	
Days PI	Baso IL-4/IL-13 (-)	Baso IL-4/IL-13 (+)
4	0.0001 ***	<0.0001 ****
6	0.0036 **	0.029 *
8	0.268 ns	0.2923 ns
10	0.1848 ns	0.843 ns

P Relative to uninfected mice

	P Value	
Days PI	Baso IL-4/IL-13 (-)	Baso IL-4/IL-13 (+)
4	<0.0001 ****	0.0019 **
6	<0.0001 ****	0.0022 **
8	0.0128 *	0.0264 *
10	0.0114 *	0.0187 *

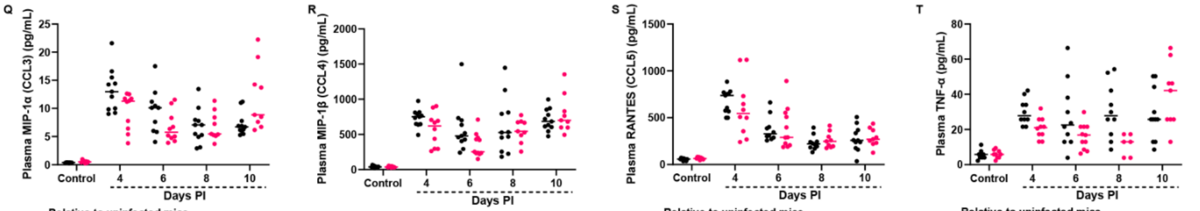

Q Relative to uninfected mice

	P Value	
Days PI	Baso IL-4/IL-13 (-)	Baso IL-4/IL-13 (+)
4	<0.0001 ****	0.0001 ***
6	0.0005 ***	0.0006 ***
8	0.0004 ***	0.0008 ***
10	<0.0001 ****	0.0038 **

R Relative to uninfected mice

	P Value	
Days PI	Baso IL-4/IL-13 (-)	Baso IL-4/IL-13 (+)
4	<0.0001 ****	0.0007 ***
6	0.0042 **	0.0012 **
8	0.0073 **	0.0002 ***
10	<0.0001 ****	0.0004 ***

S Relative to uninfected mice

	P Value	
Days PI	Baso IL-4/IL-13 (-)	Baso IL-4/IL-13 (+)
4	<0.0001 ****	0.0043 **
6	0.0004 ***	0.0073 **
8	0.0006 ***	0.0013 **
10	0.0034 **	0.0015 **

T Relative to uninfected mice

	P Value	
Days PI	Baso IL-4/IL-13 (-)	Baso IL-4/IL-13 (+)
4	<0.0001 ****	0.0001 ***
6	0.0557 ns	0.0102 *
8	0.0068 **	0.4905 ns
10	0.004 **	0.0061 **

Supplementary Figure 3. Plasma cytokines from *P. y. yoelii* 17XNL-infected Baso IL-4/IL-13 (-) and Baso IL-4/IL-13 (+) mice and uninfected controls. the y axis represents the concentrations Data were analyzed using Brown-Forsythe and Welch ANOVA tests followed by Dunnett's multiple comparisons test between infected mice at each time point and uninfected controls, or between Baso IL4/IL13 (-) and Baso IL4/IL13 (+) mice at each time point. Each dot represents a single mouse, bars correspond to the mean. P values ≤ 0.05 were considered significant. *P≤0.05, **P≤0.01, ***P≤0.001, ****P≤0.0001, ns: not significant. Black dots (Baso IL-4/IL-13).
